# The Diagnosis of Human Neurological Infection Caused by Rabies Virus Using Metagenomic Next‐Generation Sequencing: Two Case Reports

**DOI:** 10.1155/crdi/1910139

**Published:** 2026-07-01

**Authors:** Liu Yu, Zhang Chong, Li Yanchun, Hu Yingying

**Affiliations:** ^1^ Clinic Medical College, Henan University of Science and Technology, Luoyang, Henan, China, haust.edu.cn; ^2^ Department of Emergency, The First Affiliated Hospital of Henan University of Science and Technology, Luoyang, Henan, China, haust.edu.cn

**Keywords:** incubation period, mNGS, rabies, rabies virus

## Abstract

The rabies virus (RABV) causes acute progressive and fatal encephalomyelitis. Two case studies of RABV neurological infection identified using metagenomic next‐generation sequencing (mNGS) are presented in this paper. A total of 39 RABV sequences were detected using mNGS in the cerebrospinal fluid (CSF) in Case 1. The detected sequences were located in the 0%–35% range of the enriched and amplified region and had a 27 × sequencing depth. A total of 75 RABV sequences were detected using mNGS in the CSF in Case 2. These cases illustrate that mNGS use during the early diagnosis of infectious diseases is critical. They also indicate that RABV can remain latent in the human body for many years. Disease prevention education for people who have experienced bites or scratches by rabid animals is therefore crucial.

## 1. Introduction

Acute progressive and fatal encephalomyelitis is caused by the rabies virus (RABV). RABV is a single‐stranded RNA virus that belongs to the *Lyssavirus* genus. It is typically transmitted through the bite of an infected mammal [1, 2]. It also can be spread by nonbite exposures (e.g., contamination of mucous membranes or broken skin with saliva) or tissue and solid organ transplantation [3–6].

The symptoms include hypersalivation, difficulty swallowing, and hydrophobia [7]. The incubation period is typically between 20 and 90 days, but it may be as short as only a few days. It also may last for years. There are case studies which had been reported RABV lasting for more than 6 years in the human body before rabies’s symptoms occur, with supportive genetic or antigenic data [8–10]. Some reports hypothesize that longer incubation periods may occur (more than 20 years) [11–13]. However, the possibility of re‐exposure cannot be excluded. Humans will eventually die once the symptoms of rabies begin. Therefore, mNGS application for the diagnosis of rabies can be extremely helpful. An early rabies diagnosis through the use of mNGS allows for effective therapeutic measures to be taken after exposure to reduce the rabies death rate.

## 2. Case Report

### 2.1. Case 1

**FIGURE 1:**
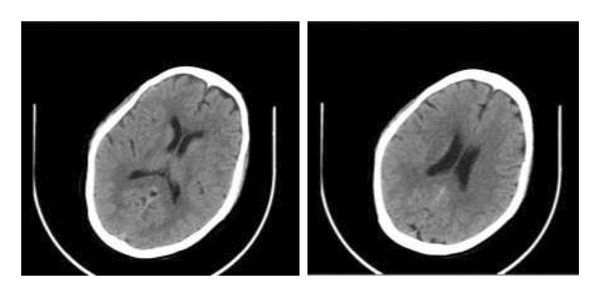
The plain CT brain imaging on the 9th day after initial symptoms.

**FIGURE 2:**
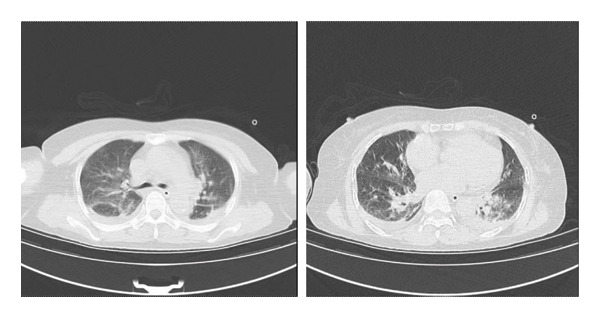
The chest CT on the 9th day after initial symptoms.

**FIGURE 3:**
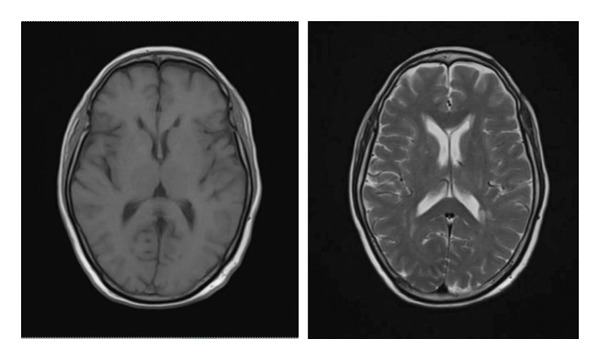
The cranial MRI on the 10th day after initial symptoms.

**FIGURE 4:**
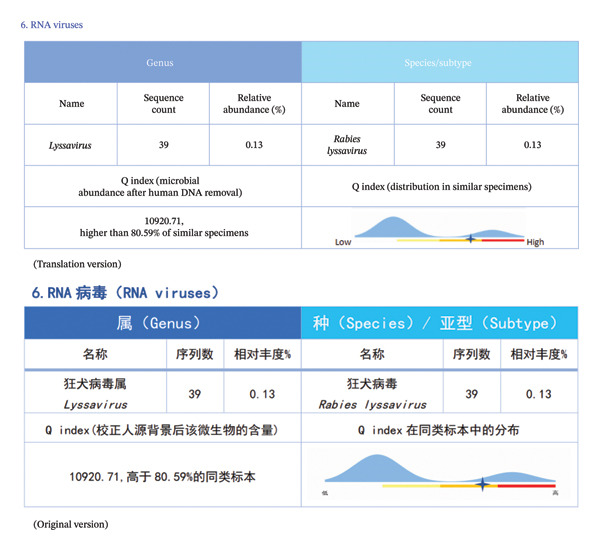
The result of mNGS about patient’s CSF on the 10th day after initial symptoms.

**FIGURE 5:**
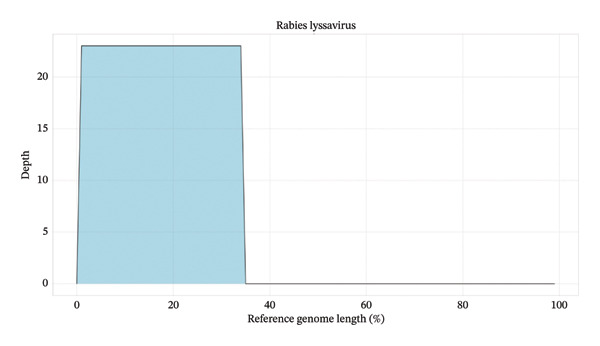
The coverage enrichment of RABV in mNGS detection about patient’s CSF.

A 70‐year‐old female farmer from Henan Province, China, was admitted to the Emergency Intensive Care Unit (EICU) of The First Affiliated Hospital of the Henan University of Science and Technology due to recurrent nausea and emesis for a duration of nine days and consciousness disturbance for seven days. The patient suddenly experienced the onset of nausea, vomiting, and consciousness disorder with no obvious cause. The initial symptoms (Day 1) were nausea, emesis, palpitations, and intermittent epigastralgia located under the ensiform appendix that was aggravated after eating and not relieved after rest. She was admitted to the local clinic and received treatment. However, her condition did not improve. After the initial symptoms, on Day 3, she developed neurological symptoms consisting of dysphoria, and she could not answer questions correctly. She was then admitted to two local hospitals for further treatment. A lumbar puncture revealed no obvious abnormalities. An upper abdomen computed tomography (CT) showed that there were some exudative changes in the bilateral perirenal areas, and her bilateral adrenal values increased. The biochemical examination showed a lactate dehydrogenase (LDH) of 297 U/L, creatine kinase (CK) of 547 U/L, hydroxybutyric dehydrogenase (HBD) of 266 U/L, potassium (K) of 3.9 mmol/L, sodium (Na) of 131.5 mmol/L, chloride (Cl) of 93.0 mmol/L, calcium (Ca) of 1.86 mmol/L, C‐reactive protein (CRP) of 50.6 mg/L, and brain natriuretic peptide (BNP) of 5122 ng/L. The cerebrospinal fluid (CSF) analysis showed a glucose level of 5.6 mmol/L, an LDH of 42 U/L, and an aspartate aminotransferase (AST) level of 36 U/L. The routine blood examination showed white blood cells (WBC) of 16.36 ∗ 10^9^/L, neutrocytes (NEU) of 14.44 ∗ 10^9^/L, a neutrophil ratio (NEU%) of 88.20%, a hemoglobin (Hb) of 113.00 g/L, and a platelet count of 159.00 ∗ 10^9^/L. Her WBC, NEU, and CRP levels were higher than normal. The patient’s symptom test results indicated the presence of central nervous system infection. Her serum sodium, chloride, and calcium levels were all lower than normal, and these may have been the cause of vomiting. In addition, her B‐type natriuretic peptide (BNP) level significantly increased, possibly because her cardiac function was impaired at that time. She was referred to our hospital on Day 9 because the previous treatment did not produce a change in symptoms. The patient experienced convulsions upon admission, but her plain brain CT remained normal (Figure 1). The chest CT showed that both lung fields had penetration, bilateral pleural effusion, and density changes in the aorta and coronary arteries (Figure 2). She had a body temperature of 37.7°C, a heart rate of 122 beats per minute, a blood pressure of 168/99 mmHg (1 mmHg = 0.133 kPa), an oxygen saturation of 97% (high‐flow oxygen inhalation constantly), an Acute Physiology and Chronic Health Evaluation II (APACHE II) score of 23, and respiratory failure. Both pupils were 3.5 mm in diameter and sensitively reactive to light. Neck stiffness, Kernig’s signs, hyperreflexia, and Hoffman’s signs, as well as the Babinski signs were not detected. A routine blood examination showed high leukocyte and neutrophil counts. In addition, the CRP was higher than normal. A neurological infection was considered on admission due to the combination of the patient’s nausea, vomiting, palpitations, delusions, convulsions, and fever. She was treated with intensive care that consisted of continuous high‐flow oxygen inhalation, heparin sodium (625 IU Q24 h), ceftriaxone sodium (4 g Q24 h), a Xing Nao Jing injection (20 mL Q24 h), phenobarbital (0.1 g Q8 h), acyclovir (0.25 g Q8 h), remimazolam Besylate (5 mg), fluid therapy, and nutritional support. Under consistent high‐flow oxygen inhalation, her blood gas analysis revealed a pH value of 7.36, carbon dioxide partial pressure of 70 mmHg, oxygen partial pressure of 69 mmHg, oxygen saturation of 93%, and lactic acid of 1.30 mmol/L. Therefore, respiratory failure was considered. Emergency orotracheal intubation and ventilator‐assisted ventilation were then administered. She lost consciousness on Day 10. Cranial magnetic resonance imaging (MRI) showed that there were multiple infarcts, encephalomalacia, and demyelination‐like changes in white matter (Figure 3). This detection did not explain the patient’s symptoms. mNGS was then conducted on the patient’s CSF. The mNGS detection of her CSF showed that there were RABV sequences (Figure 4). The mNGS detected 39 RABV sequences in the CSF, and the detected sequences were primarily located in the 0%–35% range of the enriched region, with a 27 × sequencing depth (Figure 5). The family was asked again if the patient was bitten by an animal. Her family said that the patient was bitten by a dog 20 years ago. We then confirmed that the CNS infection was caused by rabies. The patient was treated with private isolation and light avoidance. The other therapies were administered as before. There was no improvement in the patient’s condition, and she had a temperature of 36.9°C, a heart rate of 81 beats per minute, a blood pressure of 146/81 mmHg, and ventilator‐assisted breathing (P‐AC mode: FiO_2_ of 40% at 13 times per minute, PEEP of 5 cm H_2_O, and PS of 15 cm H_2_O). The patient’s prognosis was extremely poor, and her family voluntarily discharged her to home care.

### 2.2. Case 2

**FIGURE 6:**
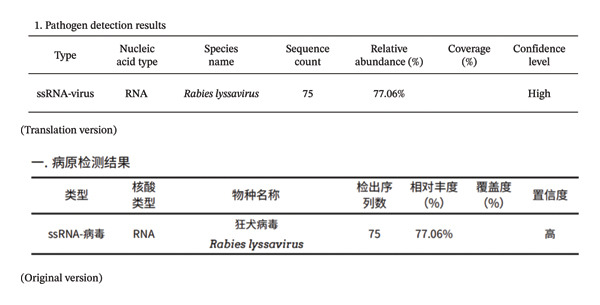
The result of mNGS about patient’s CSF on the 6th day after initial symptoms.

A 34‐year‐old female patient from Henan Province, China, with nausea and vomiting for a duration of four days and fever with hypotension for 12 h was admitted to the EICU of the First Affiliated Hospital of the Henan University of Science and Technology. The patient had rapid onset of nausea, vomiting, fever, and hypotension with no obvious cause. The initial symptoms (Day 1) were nausea, emesis, and dizziness. She was admitted to a local clinic and received treatment. Her condition did not improve. On Day 4, her nausea and emesis were worse than before. The vomit was a yellow‐white foamy liquid. She then developed new symptoms consisting of fever, hypotension, chest distress, and general fatigue. She was admitted to a local hospital for further treatment. The chest CT results revealed no obvious abnormalities, and an electrocardiogram (ECG) showed nodal tachycardia, a short PR interval, changes in the ST segment, and left ventricular hypertrophy. The myocardial infarction markers showed troponin of 3.48 ng/mL, muscle Hb of 471.10 ng/mL, and a CK isozyme of 7.65 ng/mL. Her temperature was 39.5°C. Her blood pressure was 90/60 mmHg. The test results and patient symptoms indicated the presence of septic shock. Central system infection could not be ruled out. Her myocardial enzymes were higher than normal. In addition, her ECG showed sychnosphygmia and changes in the ST segment. We concluded that her cardiac function was impaired at that time. She was referred to our hospital because the previous treatment was not satisfactory (Day 4). The patient was delirious, and her answers were irrelevant upon admission. She had a body temperature of 39.9°C, a heart rate of 156 beats per minute, a blood pressure of 109/73 mmHg, and an oxygen saturation of 99% (high‐flow oxygen was being constantly administered). Both pupils were 3.0 mm in diameter and had a sluggish light reflection. Her neck was slightly harder than normal. The entire abdomen was soft; abdominal tenderness was noted, and rebound pain was indicated. The Kernig signs, Hoffman’s signs, and the Babinski signs were not detected. Her blood routine examination showed high leukocyte and neutrophil counts. Her urine indicated positive occult blood, proteinuria at 3+, and ketonuria at 2+, with elevated levels of red and white blood cells in the urine. The blood biochemistry showed elevated liver enzymes and an electrolyte imbalance (i.e., hypokalemia and hypocalcemia). An abdominal CT scan showed multiple dilated colon sections with gas accumulation. Several portions of the small intestine were dilated with gas and fluid accumulation and air‐fluid levels. A high‐density shadow strip in the left kidney and an exudative shadow in the right inguinal area existed. An intestinal obstruction was considered, and a period of temporary fasting and symptomatic treatment was provided. The patient’s condition did not improve by Day 6, so a lumbar puncture was performed, and her CSF was sent for mNGS testing. The result showed that RABV was detected in the patient’s CSF. A total of 75 RABV sequences were detected using mNGS in the CSF (Figure 6). Upon further inquiry into the patient’s medical history, it was revealed that the patient had been bitten by an animal more than 10 years ago. However, in the absence of molecular epidemiological linkage, this historical exposure cannot be definitively established as the causative event, and other unrecognized exposures during the intervening period cannot be excluded. We treated the patient in private isolation with light avoidance. The patient suddenly experienced a heart rate decrease, slowed breathing, and decreased oxygen saturation on Day 9, suggesting multiple organ failure. Symptomatic treatments, such as cardiopulmonary resuscitation, defibrillation, endotracheal intubation, and the use of a ventilator, were administered. The patient fell into a coma on Day 10, with a large amount of secretions visible in the oral cavity. The mouth secretions significantly increased when fanned. This was accompanied by laryngeal muscle spasms and whole‐body muscle tremors, indicating positive hydrophobia and aerophobia. These symptoms were consistent with the clinical manifestations of rabies. The patient’s symptoms further progressed on Day 11, and symptomatic treatments such as ceftriaxone–sulbactam combined with vancomycin for anti‐infection and acyclovir for anti‐viral were administered. The patient’s consciousness disorder further progressed on Day 12, and she was in a deep coma; however, oral secretions decreased and muscle tremors were somewhat alleviated. The patient’s family requested her voluntary discharge due to her poor prognosis.

## 3. Discussion

RABV is a single‐stranded RNA virus that is very sensitive to ultraviolet light, high temperatures, and dry environments. RABV loses its activity at high temperatures [14]. The RABV incubation period differs. It can even lie dormant in the human body for a year or longer [8–13]. In the reported cases, the primary symptoms were fear, irritability, salivation, convulsions, and other symptoms, and these were confirmed to be caused by rabies primarily based on a history of animal injury and clinical symptoms. But the possibility of re‐exposure cannot be excluded.

The natural rabies course is incubation, a prodromal stage, an acute neurological symptom phase, a paralysis period, and death. In the first case, we knew that the patient was bitten by a dog 20 years ago. However, the incubation period for rabies varies from 1 week to 1 year and is typically one to 3 months, but rarely more than a year [15]. Hence, it cannot be determined whether there was a clear association in these case studies. Perhaps the rabid animal had licked the patient’s mucosa or injured skin, the wound contacted the rabid animal’s saliva or tissue directly, or the wound was just a light scratch without bleeding. Therefore, the patients and their families did not believe there was close contact. Hence, they could not provide an accurate medical history. Therefore, when we suspect that a patient is infected by RABV, we should not only ask whether they were scratched or bitten by mammals but also ask about nonbite exposures (e.g., contamination of mucous membranes or broken skin with saliva) or tissue and solid organ transplantation.

The prodromal phase begins with symptoms such as malaise, anorexia, fatigue, headache, and fever, with 50%–80% of patients experiencing specific neuropathic pain or paresthesia at the original exposed area typically 2–10 days after contact [14]. The analyzed cases showed symptoms of fatigue, nausea, and vomiting, but these did not attract attention. Rabies can manifest as various clinical syndromes, among which the furious and paralytic forms are the two most common types. However, its clinical manifestations exhibit significant heterogeneity, a characteristic influenced by multiple factors such as viral strain properties, route of infection, and host immune status [16, 17]. Furious cases have extreme fear, hydrophobia, wind fear, pharyngismus, dyspnea, difficulty in urination and defecation, hidrosis, and salivation. Paralytic cases typically have high fever, headache, vomiting, limb weakness, ventosity, dystaxia, and gatism. Both these cases were of the furious type where the patients had obvious symptoms of hydrophobia and fear of wind. The first patient’s family clearly denied that the patient had a history of rabies exposure. In addition, the patient was ill for 9 days, beyond the survival time after the onset of rabies [18]; hence, we excluded the possibility of rabies temporarily upon admission. In the second case, her CSF was sent for mNGS detection on the third day of admission, and the mNGS detection outcome showed that there were RABV sequences in her CSF. Prior to dying, most patients go into a coma, and respiratory arrest generally occurs shortly after coma. In our first case, the patient went into a coma on the second day of admission (the 10th day after the initial symptoms occurred), and the RABV sequences were detected in her CSF using mNGS detection. The rabies diagnosis was confirmed when combined with the clinical manifestations. In the second case, the patient went into a coma on the 7th day of admission (the 10th day after the initial symptoms occurred).

The rabies laboratory diagnostic standards are as follows: If the RABV antigen or RT‐PCR nucleic acid is positive in the patient’s saliva, CSF, or the tissue of the back of the neck covered with hair follicles; or the RABV can be isolated from the patient’s saliva or CSF; or we can detect the RABV antigen or RABV as positive in the brain tissue in the corpse; or we can isolate the RABV by cell culturing of cells from the brain tissue in the corpse, the rabies diagnosis can be confirmed [19].

The initial symptoms in these cases manifested as gastrointestinal symptoms (e.g., nausea, vomiting, and fever), which align with the early symptoms of rabies; however, due to their lack of specificity, these symptoms often fail to attract adequate attention. On the 3rd day after the initial symptoms, the first patient displayed a consciousness disorder and then went to several hospitals, but the pathogeny remained unclear. The diagnoses of these patients were difficult to determine. This may have been related to the blood biochemistry, head CT, MRI, and other test results of rabies that have no specificity. The rabies diagnosis primarily depends on the exposure history and laboratory testing methods. If the patient denies a rabies exposure history, we may not do the related tests and treatment measures for rabies. In addition, rabies laboratory testing methods have limitations. First, the negative result of a fluorescent antibody test or enzyme‐linked immunosorbent test cannot exclude rabies. Second, the cell culture method has a long cultivation cycle. Third, a brain tissue test is primarily used for an autopsy diagnosis [19]. For some unknown or rare pathogenic microorganisms, the traditional detection methods for pathogenic microorganisms cannot identify them quickly and cannot meet the monitoring needs of clinically acute and critical patients. mNGS detection obtains the types and information of microorganisms using high‐throughput sequencing of nucleic acids extracted from biological specimens. This detection can directly conduct simultaneous sequencing and analysis of nearly all microbial nucleic acids in samples, and it is an important method for microbiome research in the human body and various environments. It can be used to aid infectious disease diagnosis. This method eliminates the limitations of traditional microbiology based on culture and identification and can obtain the sequence of nearly all DNA or RNA in a sample to analyze the microbial spectrum and even the genome or transcriptome of the host [20]. It can detect more potential infectious agents in one detection without reliance on prior knowledge of the target pathogen. It is especially useful for the diagnosis of acute, critical, and difficult infectious diseases. But mNGS also has its limitations: (1) reagent‐derived nucleic acids and laboratory contamination can critically impact sequence‐based microbiome analyses [21]; (2) factors such as high costs, personnel training requirements, the degree of methodological standardization, and data analysis capabilities may influence the adoption and application scope of mNGS [22]. For patients with encephalitis of unknown etiology, while strictly adhering to standard infection control and prevention measures, the possibility of rabies must be considered [16]. Compared with traditional etiological testing, mNGS provides broader pathogen coverage and eliminates the need for prior prediction of the causative pathogen, showing significant advantages in the diagnosis of encephalitis of unknown origin. This is especially the case for infectious diseases like rabies, which have low incidence rates, nonspecific early symptoms, and low positivity rates in conventional testing. mNGS effectively improves the efficiency of early diagnosis, allowing for timely adjustment of treatment plans and avoiding unnecessary waste of medical resources [23].

## Funding

No funding was received for this manuscript.

## Consent

Written informed consent was obtained from the patient for the publication of this case report.

## Conflicts of Interest

The authors declare no conflicts of interest.

## Data Availability

The data that support the findings of this study are available on request from the corresponding author. The data are not publicly available due to privacy or ethical restrictions.
